# The Co-Stimulatory Effects of MyD88-Dependent Toll-Like Receptor Signaling on Activation of Murine γδ T Cells

**DOI:** 10.1371/journal.pone.0108156

**Published:** 2014-09-18

**Authors:** Jinping Zhang, Jia Wang, Lan Pang, Guorui Xie, Thomas Welte, Vandana Saxena, Jason Wicker, Brian Mann, Lynn Soong, Alan Barrett, Willi Born, Rebecca O'Brien, Tian Wang

**Affiliations:** 1 Department of Microbiology & Immunology, University of Texas Medical Branch, Galveston, Texas, United States of America; 2 Department of Pathology, University of Texas Medical Branch, Galveston, Texas, United States of America; 3 Integrated Department of Immunology, National Jewish Health Center, Denver, Colorado, United States of America; University of Hong Kong, Hong kong

## Abstract

γδ T cells express several different toll-like receptor (TLR)s. The role of MyD88- dependent TLR signaling in TCR activation of murine γδ T cells is incompletely defined. Here, we report that Pam3CSK4 (PAM, TLR2 agonist) and CL097 (TLR7 agonist), but not lipopolysaccharide (TLR4 agonist), increased CD69 expression and Th1-type cytokine production upon anti-CD3 stimulation of γδ T cells from young adult mice (6-to 10-week-old). However, these agonists alone did not induce γδ T cell activation. Additionally, we noted that neither PAM nor CL097 synergized with anti-CD3 in inducing CD69 expression on γδ T cells of aged mice (21-to 22-month-old). Compared to young γδ T cells, PAM and CL097 increased Th-1 type cytokine production with a lower magnitude from anti-CD3- stimulated, aged γδ T cells. Vγ1^+^ and Vγ4^+^ cells are two subpopulations of splenic γδ T cells. PAM had similar effects in anti-CD3-activated control and Vγ4^+^ subset- depleted γδ T cells; whereas CL097 induced more IFN-γ production from Vγ4^+^ subset-depleted γδ T cells than from the control group. Finally, we studied the role of MyD88-dependent TLRs in γδ T cell activation during West Nile virus (WNV) infection. γδ T cell, in particular, Vγ1^+^ subset expansion was significantly reduced in both MyD88- and TLR7- deficient mice. Treatment with TLR7 agonist induced more Vγ1^+^ cell expansion in wild-type mice during WNV infection. In summary, these results suggest that MyD88-dependent TLRs provide co-stimulatory signals during TCR activation of γδ T cells and these have differential effects on distinct subsets.

## Introduction

γδ T cells are a minority of CD3^+^ T cells in lymphoid tissue and blood of humans and rodents, but are well represented at epithelial and mucosal sites [1]. They can rapidly proliferate after parasitic, bacterial, and viral infections and produce inflammatory cytokines, such as IFN-γ and TNF-α [2]–[6]. These cells lack major histocompatibility complex (MHC) restriction and have the potential capacity to respond to antigens without a requirement for conventional antigen processing [7], [8]. Unlike αβ T cells, there are few antigens recognized by γδ T cell receptor [9]. Human Vδ2 T cells recognize small bacterial phosphoantigens, alkylamines and synthetic aminobisphosphonates; whereas Vδ1 T cells recognize stress-inducible MHC-related molecules-MICA/B and other ligands [10], [11]. The class I molecules, including class Ib, and CD1d, are ligands for some murine γδ T cells [12]–[14]. In addition, both murine and human γδ T cells recognize the algae protein phycoerythrin [15]. Taken together, these unique features suggest that γδ T cells play a role in innate immunity during microbial infection. However, the underlying immune mechanisms of γδ T cell activation are not clearly understood.

Toll-like receptors (TLRs) play a fundamental role in host innate immunity by mounting a rapid and potent inflammatory response to pathogen infection by their recognition of conserved structural patterns in diverse microbial molecules. They are expressed by a wide range of cells, including both immune cells and non-immune cells. The core TLR signaling pathway (except for TLR3) utilizes myeloid differentiation factor 88 (MyD88) as the primary adaptor [16]–[18]. Mouse and human γδ T cells express TLR2, TLR3, TLR4 and TLR7/8 [19]–[23]. Some studies suggest TLR-mediated signaling pathways can indirectly activate γδ T cells, mainly via cross- talk between these cells and dendritic cells (DCs) [24]–[27]. For human γδ T cells, TLR ligands are also known to co-stimulate TCR-activation. For example, TLR2, TLR3 and TLR5 ligands induced higher cytokine production and increased expression of cell surface activation markers [28]–[30]. However, the direct effect of TLR ligands on activation of murine γδ T cells is not clearly defined. In this study, we investigated the role of MyD88-dependent TLRs in activating murine γδ T cells.

## Materials and Methods

### Mice

6-to 10-week-old control C57BL/6 (B6), TLR4 deficient (TLR4^−/−^) mice and 21-to 22-month-old B6 mice were purchased from Jackson Laboratories (Bar Harbor, ME) and the National Institute of Aging (Bethesda, MD), respectively. MyD88^−/−^ mice were bred to the B6 background by backcrossing for 10 successive generations [31], [32]. TLR7^−/−^ mice (B6×129 F_2_ background) were obtained from Regeneron Inc. (Tarrytown, NY) and bred to the B6 background by backcrossing for 7 successive generations [33], [34]. Groups were age- and sex-matched for each experiment and housed under identical conditions. This study was carried out in strict accordance with the recommendations in the Guide for the Care and Use of Laboratory Animals of the National Institutes of Health. All animal experiments were approved by the Animal Care and Use Committee at the University of Texas Medical Branch (Permit #0902011).

### Stimulation of γδ T cells with anti-CD3 with or without TLR agonists

γδ T cells were purified from the pooled spleens of 3–5 mice by using a TCRγ/δ^+^ T Cell Isolation Kit according to the manufacturer's instructions (Miltenyi Biotec, Auburn, CA). The purity of γδ T cells was examined by staining with streptavidin-PE and anti-CD3 FITC. γδ T cells (1×10^5^ cells/well) were cultured for 2 days at 37°C in RPMI-1640 medium (Invitrogen, Carlsbad, CA) in 96-well plates coated with 5 µg/ml anti-CD3 (eBioscience, San Diego, CA) in the presence of 1 µg/ml of Pam3CSK4 (PAM, Invivogen, San Diego, CA), or 10 µg/ml of lipopolysaccharide (LPS, Sigma, St. Louis, MO) or 1 µg/ml of CL097 (Invivogen). At 48 h post-treatment, cells were harvested and stained for cell surface markers. Culture supernatant was harvested for measurement of cytokine production.

### Flow cytometry

Cells were stained with monoclonal antibody (mAb) GL3-FITC (hamster anti-mouse TCRδ, BD Biosciences, San Diego, CA), and/or Abs for CD3, CD25, and CD69 (e-Bioscience). In some experiments, γδ T cells were labeled with 2.5 µM carboxyfluorescein succinimidyl ester (CFSE) according to the manufacturer's instructions (Invitrogen) and cultured at 1×10^5^ cells/well for 48 h. γδ T cell proliferation was assessed by flow cytometric analysis of CFSE dilution. After staining, cells were fixed with 0.5% paraformaldehyde in PBS and examined by using a C6 Flow Cytometer (Accuri cytometers, Ann Arbor, MI). To study TLR7 expression, cells were stained with Abs for Vγ1, or Vγ4, fixed in 2% paraformaldehyde, and permeabilized with 0.5% saponin before adding PE-conjugated anti-TLR7 or control IgG (Thermo Scientific Pierce, Waltham, MA). Data were analyzed by using CFlow Plus (Accuri cytometers).

### *In vivo* depletion of γδ subpopulations

Vγ4 T-cell depletion was achieved by two consecutive injections of 100 µg of hamster anti-Vγ4 (mAb UC3, purified from hybridoma culture supernatants [35] intraperitoneally (i.p.). at 2 days and 24 h before mice were euthanized for tissue harvesting [36], [37]. Sham Ab treatments were performed with the same amount of hamster IgG isotype (Innovative Research, Southfield, MI).

### Cytokine assays

Culture supernatant was collected for analysis of cytokine production by using a Bio-Plex Pro Mouse Cytokine Assay (Bio-Rad, Hercules, CA) or an ELISA (BD Bioscience).

### West Nile virus (WNV) infection in mice

The WNV NS4B-P38G attenuated mutant infectious clone-derived virus [38] was passaged once in Vero cells to make a virus stock for infection studies. Mice were inoculated i.p. with 1500 plaque forming units (PFU) of WNV NS4B-P38G mutant. In some experiments, mice were injected i.p. with 30 µg of R848 (R848 VacciGrade, Invivogen) [39] 4 h before WNV infection. At various time points post-infection, splenocytes were harvested from WNV- infected mice and non-infected controls and stained for CD3 and TCRγδ.

### Statistical analysis

Data analysis was performed by using Prism software (Graph-Pad) statistical analysis. Values for phenotype analysis and cytokine production experiments were presented as means ± SEM. *P* values of these experiments were calculated with a non-paired Student's t test or Mann-Whitney test. Statistical significance was accepted at *P*<0.05.

## Results

### TLR2 and TLR7, but not TLR4 agonists act in synergy with anti-CD3 treatment in the activation of murine γδ T cells *in vitro*

**Figure 1:**
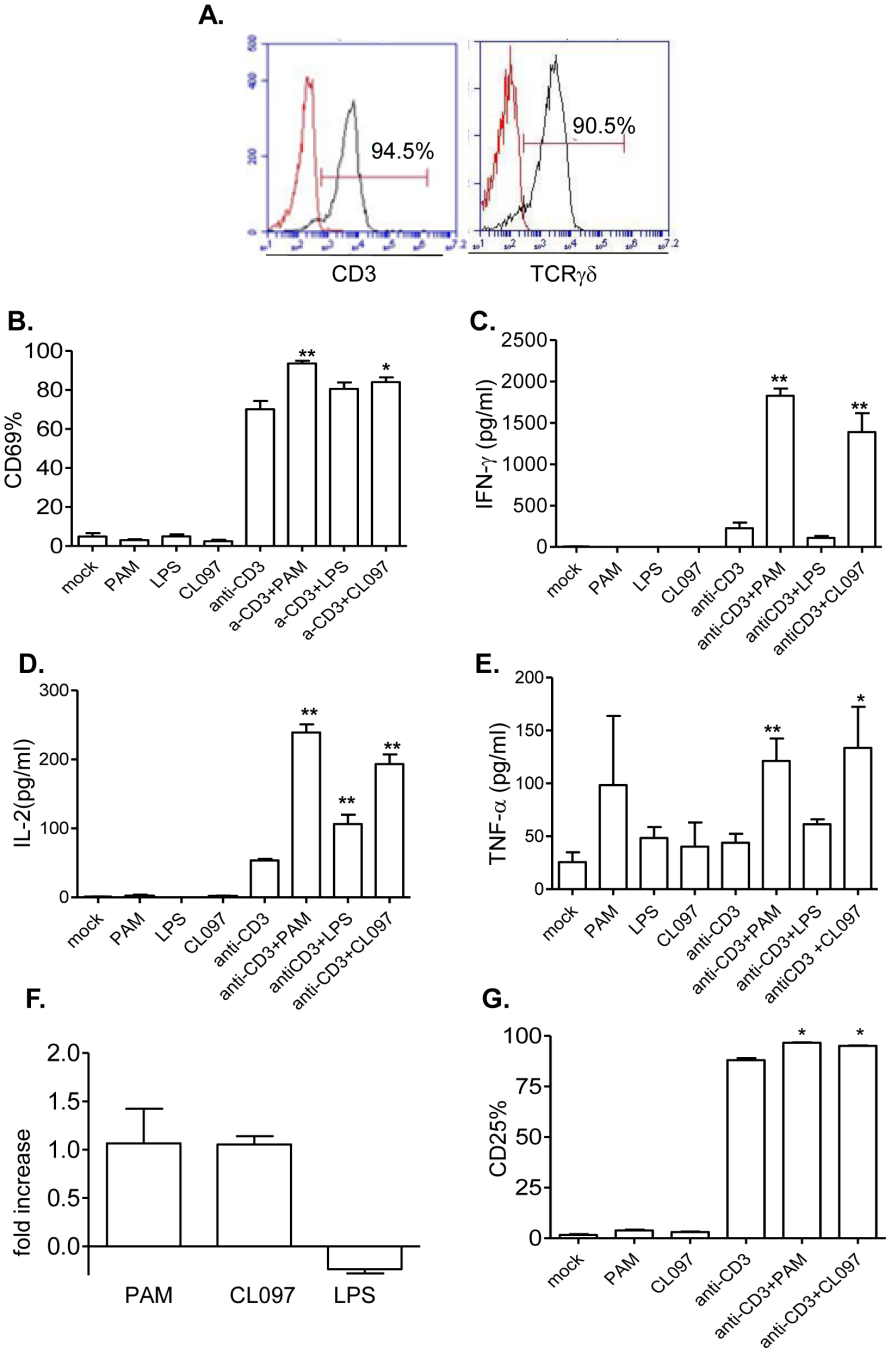
The effects of TLR2, 4 and 7 agonists on anti-CD3- treated murine γδ T cells. *A*, Flow cytometry analysis of splenic γδ T cells stained with antibodies to TCRγδ and CD3. *B–E*, splenic γδ T cells were cultured with anti-CD3 with or without TLR agonists. Cells were harvested at 48 h post-stimulation and examined for CD69 expression (*B*), and IFN-γ (*C*), IL-2 (*D*) and TNF-α (*E*) production in culture supernatant. *F*. *In vitro* T cell proliferation assay. CFSE- labeled γδ^+^ T cells were cultured for 48 h in the presence of anti-CD3 with or without TLR agonists. Data shown are fold of increase of T cell proliferation compared to anti-CD3 treated cells. *G*. CD25 expression. Data are presented as means ± SEM, *n* = 4–7. ** *P*<0.01 or * *P*<0.05 compared to anti-CD3- treated cells. Data presented are one representative of at least four similar experiments.

Murine γδ T cells express TLR2, TLR3, TLR4 and TLR7 [18], [21]. Here, we examined the role of MyD88-dependent TLR ligands in activating murine γδ T cells. We isolated splenic γδ T cells of B6 mice by magnetic beads. Flow cytometry analysis showed that the purified cells were more than 90% positive for CD3 or TCRγδ (**Figure 1A**). These cells were next treated with anti-CD3 and TLR agonists, including PAM (Pam3CSK4, TLR2 ligand), LPS (TLR4 ligand) or CL097 (TLR7 ligand). At 48 h after the treatment, we detected about 70% upregulation of the early activation marker- CD69 expression on anti-CD3-stimulated γδ T cells (**Figure 1B**). PAM, LPS or CL097 alone did not induce CD69 expression. However, PAM and CL097 increased CD69 expression by 33% or 20% respectively, when used together with anti-CD3 (*P*<0.01 or *P*<0.05). LPS did not have the same effect on CD69 expression (Figure 1B). We also measured T- helper 1 (Th1) -type cytokine production in cell culture supernatant. Both PAM and CL097 synergized with anti-CD3 in inducing IFN-γ, IL-2 and TNF-α production from γδ T cells (*P*<0.01 or *P*<0.05); whereas LPS did not have the same effect, except in the study with anti-CD3-treated γδ T cells from which IL-2 was produced (**Figure 1C–E**, *P*>0.05). Furthermore, none of the TLR agonists could induce γδ T cell proliferation by itself (data not shown). However, PAM and CL097, but not LPS increased γδ T cell number following treatment with anti-CD3 (**Figure 1F**). Concurrent with these findings, PAM and CL097 also enhanced CD25 expression on anti-CD3-treated γδ T cells (**Figure 1G**, *P*<0.05).

**Figure 2:**
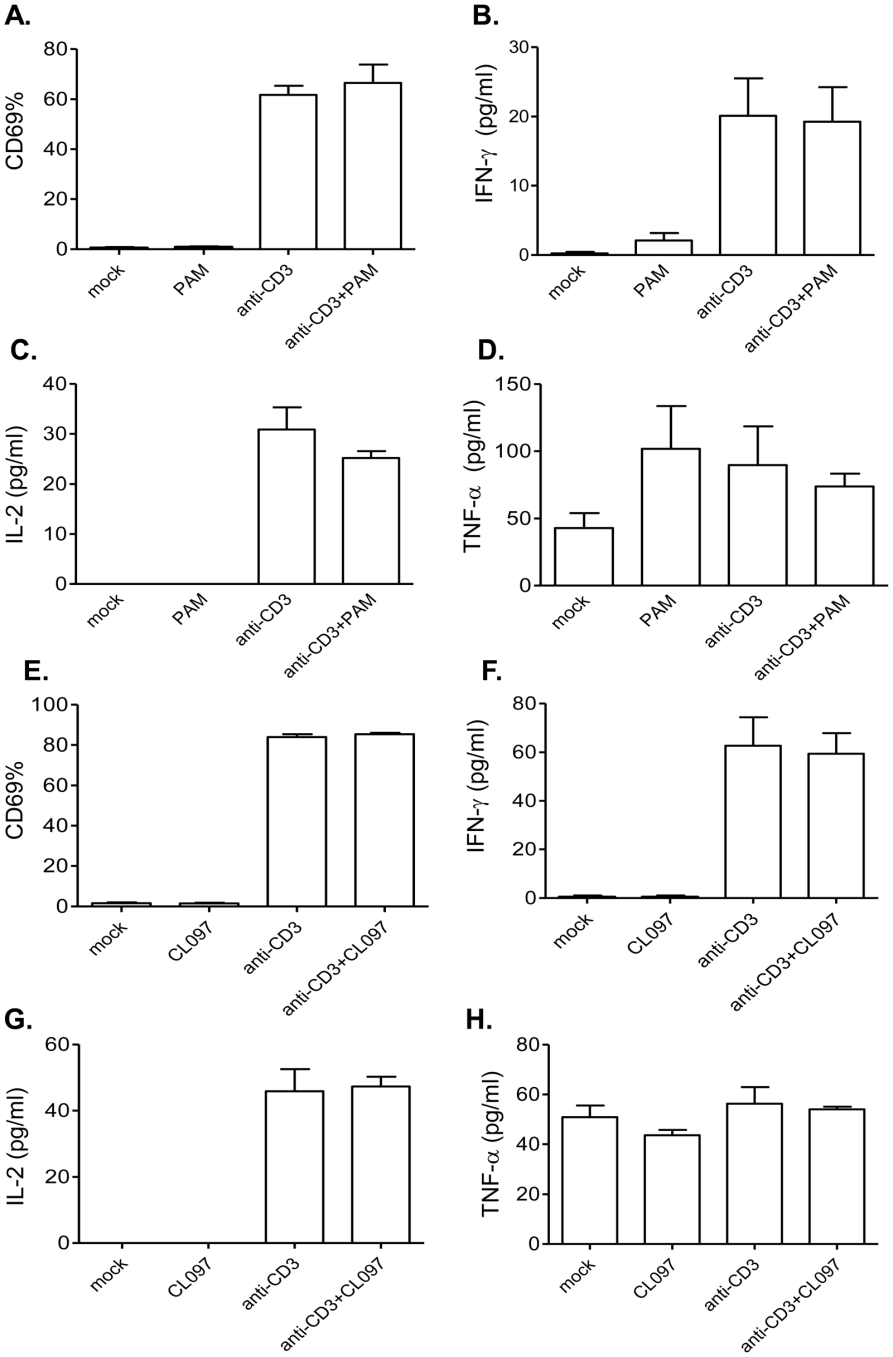
The effects of TLR2 and TLR7 ligands on anti-CD3- activated γδ T cells isolated from MyD88^−/−^ and TLR7^−/−^ mice. Splenic γδ T cells of MyD88^−/−^ (*A*–*D*) or TLR7^−/−^ mice (*E*–*H*) were cultured with anti-CD3 with or without TLR agonists. Cells were harvested at 48 h post-stimulation and analyzed for CD69 expression (*A*) & *(E)* and IFN-γ (*B*) & (*F*), IL-2 (*C*) & (*G*) and TNF-α (*D*) & (*H*) production in culture supernatant. Data are presented as means ± SEM, *n* = 3–4. ** *P*<0.01 or * *P*<0.05 compared to anti-CD3- treated cells. Results presented are one representative of three similar experiments.

To verify these findings, we next measured CD69 expression and Th-1 type cytokine production from γδ T cells of MyD88^−/−^ or TLR7^−/−^ mice following 48 h of stimulation with anti-CD3 and PAM or CL097. As found in wild-type γδ T cells, anti-CD3 induced CD69 expression and Th-1 type cytokine production from γδ T cells isolated from MyD88^−/−^ (**Figure 2A–D**) or TLR7^−/−^ mice (**Figure 2E–H**). Neither PAM nor CL097 had synergistic effects with anti-CD3 in inducing the expression of CD69 (**Figures 2A & 2E**, *P*>0.05) and IFN-γ, IL-2 and TNF-α production (**Figures 2B–D & 2F–H**, *P*>0.05). Furthermore, LPS treatment had the same effect on anti-CD3-activated γδ T cells of TLR4^−/−^ mice, compared to that on wild-type γδ T cells (**Figure S1**, *P*>0.05). These data suggest that TLR2 and TLR7 act as co-stimulatory factors during *in vitro* TCR-activation of murine γδ T cells.

### Effects of TLRs 2 and 7 agonists on the activation of γδ T cells from aged mice

**Figure 3:**
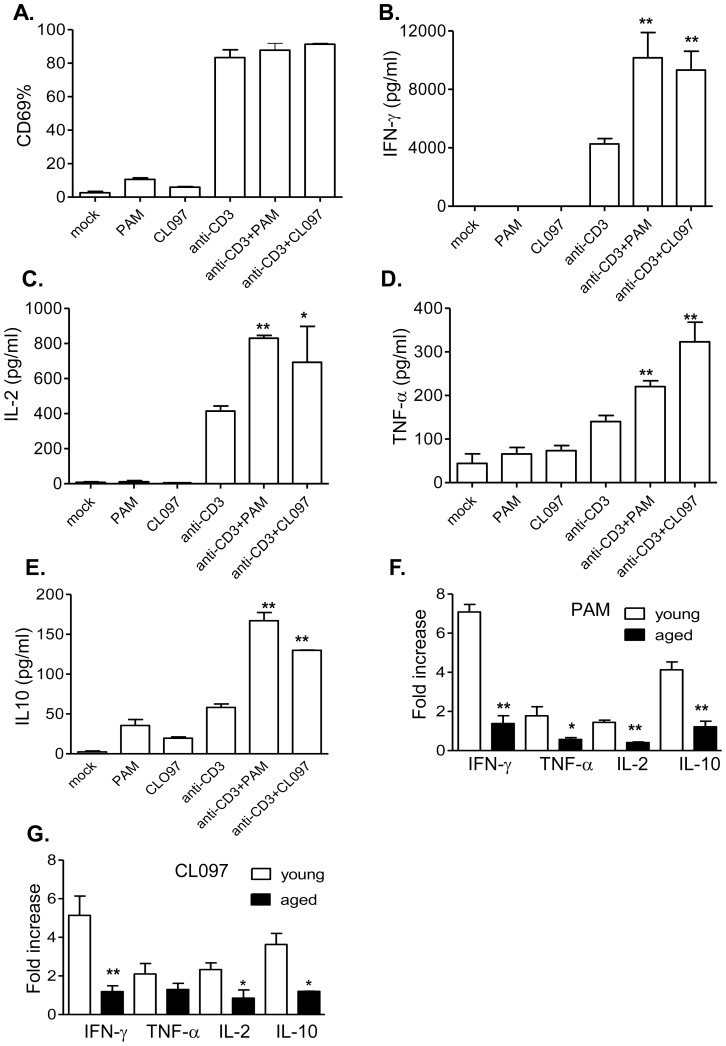
The effects of TLR2 and TLR7 ligands on anti-CD3- activated γδ T cells of aged mice. Splenic γδ T cells isolated from aged mice were cultured with anti-CD3 with or without TLR agonists. Cells were harvested at 48 h post-stimulation and analyzed for CD69 expression (*A*) and the production of IFN-γ (*B*), IL-2 (*C*), TNF-α (*D*) and IL-10 (*E*) in culture supernatant. Data are presented as means ± SEM, *n* = 3–8. ** *P*<0.01 or * *P*<0.05 compared to anti-CD3- treated alone. *F–G*. Fold of increase of cytokine production by TLR2 *(E)* or TLR7 agonists *(F)* of young and aged γδ T cells compared to anti-CD3 treated alone. ** *P*<0.01 compared to young γδ T cells.

Age-associated dysregulation of TLR signaling has been reported to contribute to the increased morbidity and mortality from infectious diseases found in geriatric patients [40], [41]. γδ T cells are also known to display numerical and functional alteration with aging [37], [42]–[44]. Nevertheless, the role of TLRs in dysfunction of aged γδ T cells is not well understood. Here, we isolated γδ T cells from aged (21-to 22-month-old) B6 mice and stimulated them with anti-CD3 and TLRs 2 and 7 agonists. Although anti-CD3 induced CD69 expression on aged γδ T cells, neither PAM nor CL097 increased CD69 expression when treated with anti-CD3 (**Figure 3A**, *P*>0.05). PAM or CL097 enhanced the production of IFN-γ, IL-2, and TNF-α from anti-CD3 stimulated aged γδ T cells (**Figure 3B–3D**, *P*<0.01 or *P*<0.05). We also measured the production of regulatory cytokines, including IL-10 and TGF-β. Both PAM and CL097 increased the production of IL-10 following anti-CD3 treatment on aged γδ T cells (**Figure 3E**, *P*<0.01). No changes in TGF-β levels were noted following any of the treatments on γδ T cells (data not shown). Furthermore, the magnitude of the synergistic effects of TLR agonists with anti-CD3 was much lower compared to that in young γδ T cells (**Figure 3F & 3G**, *P*<0.05 or *P*<0.01), except for induction of TNF-α production following CL097 and anti-CD3 treatment (**Figure 3G**, *P*>0.05). These results indicate that TLR2 and TLR7 have reduced co-stimulatory effects on activating γδ T cells of aged mice.

### Effects of TLRs 2 and 7 agonists on activation of γδ T cells in the absence of Vγ4^+^ subsets

**Figure 4:**
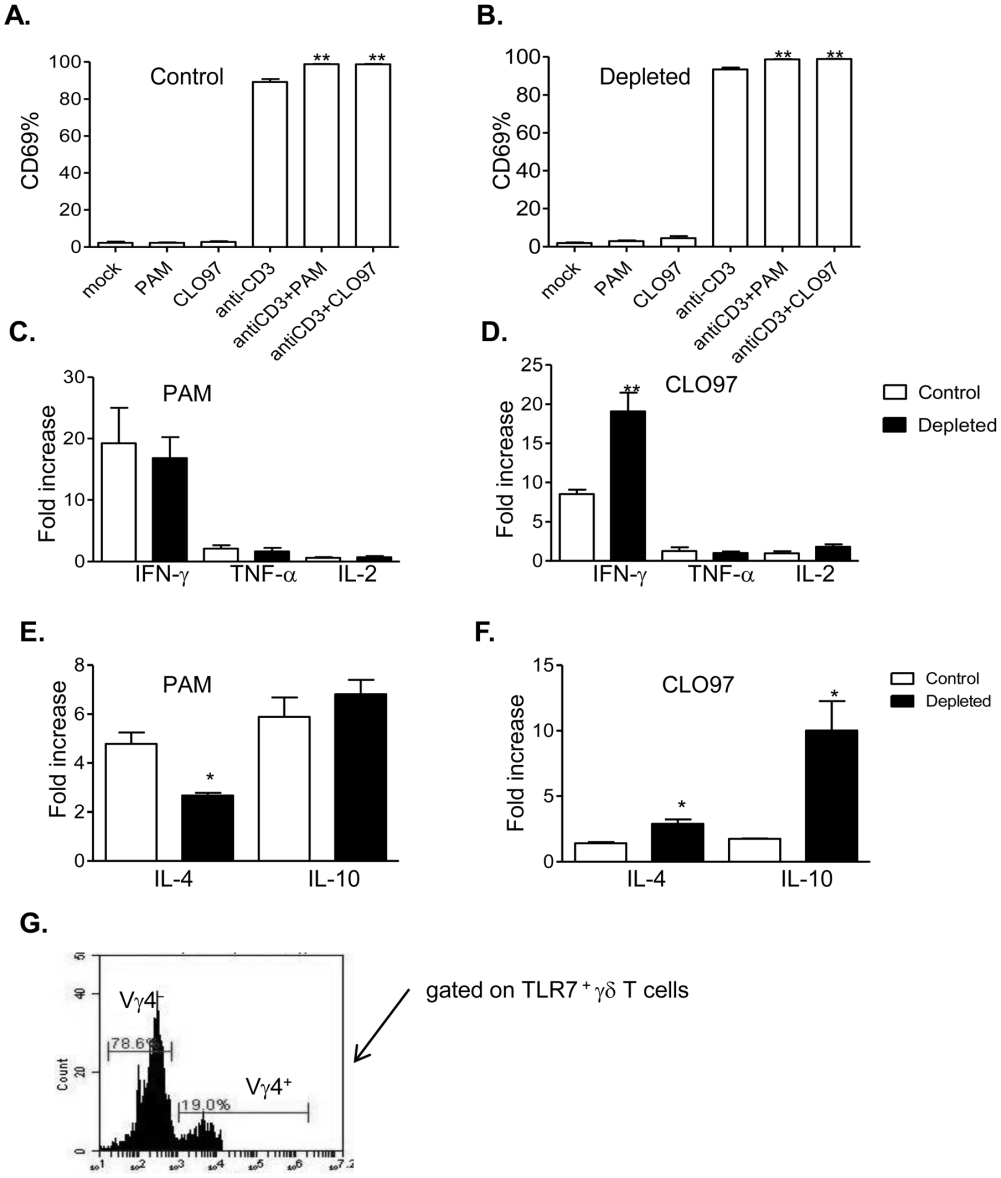
The effects of TLR2 and TLR7 ligands on anti-CD3- activated γδ T cells depleted of Vγ4^+^ subsets. Splenic γδ T cells isolated from mice depleted with control IgG or antibody to Vγ4^+^ were cultured with anti-CD3 with or without TLR agonists. Cells were harvested at 48 h post-stimulation and analyzed for CD69 expression. *A*. Control group. *B*. Vγ4^+^ cell-depleted γδ T cells. *C*–*F*. Fold of increase of Th-1 cytokine, IL-4 and IL-10 production by TLR2 *(C–E)* or TLR7 agonists *(D–F)* of Control and Vγ4^+^ cell-depleted γδ T cells compared to anti-CD3 treated alone. ** *P*<0.01 compared to young mice. ** *P*<0.01 or * *P*<0.05 compared to anti-CD3- treated alone. *G*. γδ T cells were stained with Abs for γδ T cell subsets and TLR7. TLR7-positive cells were gated for phenotypic analysis of γδ T cell subsets. ** *P*<0.01 or * *P*<0.05 compared to Vγ4^+^ cell-depleted γδ T cells. Results presented are one representative of three similar experiments.

γδ T cells are divisible into functionally distinct subsets, which distribute in an organ-specific manner [45]. Vγ1^+^ and Vγ4^+^ T cells represent two major populations of splenic γδ T cells in B6 mice [37]. We evaluated the effects of TLRs 2 and 7 agonists on splenic Vγ4^+^ T cell activation. Mice were first depleted with control IgG or Vγ4^+^ antibodies, as described before [36], [37]. Next, γδ T cells were isolated and stimulated *in vitro* with anti-CD3 and TLR agonists. Both PAM and CL097 increased CD69 expression on anti-CD3-treated controls and Vγ4^+^ cell-depleted γδ T cells (**Figure 4A & 4B**, *P*<0.01 or *P*<0.05). Further, PAM enhanced the production of all three cytokines from anti-CD3 stimulated cells in both groups in a similar manner (**Figure 4C**, *P*>0.05). Compared to the control group, CL097 induced more IFN-γ production from anti-CD3 activated -γδ T cells that were depleted of Vγ4^+^ cells (**Figure 4D**, *P*<0.01). To determine the regulatory role of Vγ4^+^ T cells, we next measured IL-4 and regulatory cytokine production. While no changes in TGF-β expression was noted following the treatment (data not shown), PAM induced less IL-4 production from anti-CD3 treated -Vγ4^+^ cell-depleted γδ T cells compared to the control group (**Figure 4E**, *P*<0.05). CL097 treatment induced more IL-4 and IL-10 production from Vγ4^+^ cell-depleted cells following anti-CD3 stimulation (**Figure 4F**, *P*<0.05). Finally, we determined TLR7 expression on the two splenic γδ T cell subsets. We found that nearly 80% of TLR7-positive γδ T cells were Vγ4^−^; while only 20% were Vγ4^+^ cells (**Figure 4G**). Thus, these results indicate that the differences in TLR7 expression among γδ T cell subsets contribute to a differential co-stimulatory effect of TLR7 agonist on these cells.

### Role of TLR7 in γδ T cell activation and expansion in response to WNV infection

**Figure 5:**
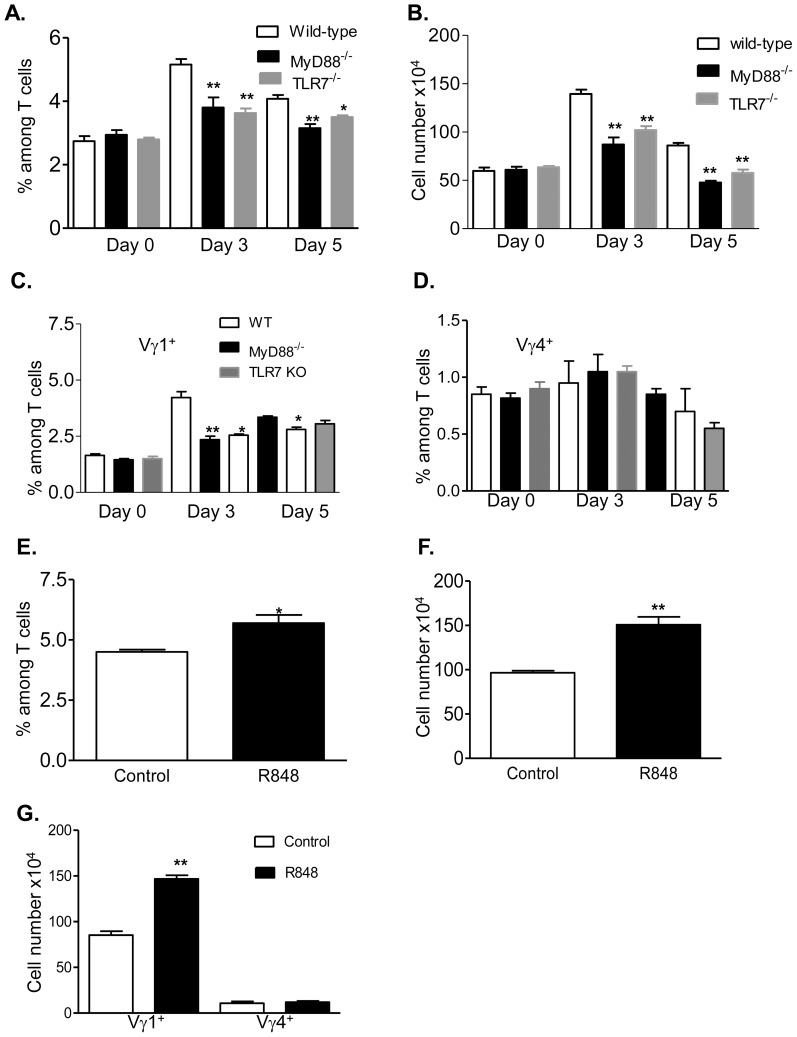
TLR7-mediated γδ T cell activation during WNV infection. Splenic T cells of wild type, MyD88^−/−^ and TLR7^−/−^ mice were isolated before infection (day 0) and at days 3 and 5 post-infection and stained for TCRγδ, Vγ1^+^, Vγ4^+^ and CD3. ** *P*<0.01 or * *P*<0.05 compared to wild-type mice. *A*. Total γδ^+^ percentage among splenic T cells. *B*. Splenic γδ cell number per mouse. *C–D*. Vγ1^+^ (*C*) or Vγ4^+^ (*D*) percentage among splenic T cells. *E*–*G*. Wild-type B6 mice were injected with R848 followed by infection with WNV NS4B-P38G mutant. Cells were harvested at day 3 post-infection and stained for TCRγδ, Vγ1^+^, Vγ4^+^ and CD3. *E*. Total γδ^+^ percentage among splenic T cells. *F*. Splenic γδ cell number per mouse. *G*. Total number of Vγ1^+^ and Vγ4^+^ cells per mouse. ** *P*<0.01 or * *P*<0.05 compared to control group.

WNV is a mosquito-borne flavivirus with a positive-sense, single-stranded RNA genome. Following wild-type WNV infection, γδ T cells expand significantly in mice, produce IFN-γ and protect the host from lethal encephalitis [46]. TLR7 is required for host protective immunity during wild-type or the attenuated WNV NS4B-P38G mutant infection [32], [34], [47]. To determine the role of MyD88-dependent TLR signaling in γδ T cell activation during WNV NS4B-P38G mutant infection, splenocytes were isolated and assessed before infection (day 0) and at early (day 3) and later (day 5) intervals post infection. The percentage of γδ T cells in wild-type mice increased significantly at day 3 post infection, and decreased though remained higher than non-infected controls at day 5 post-infection. The percentage of γδ T cells was also increased in MyD88^−/−^or TLR7^−/−^ mice at day 3, but became not significant at day 5 following infection (**Figure 5A**, *P*>0.05). In comparison to wild-type mice, the magnitude of γδ T cell expansion at day 3 post-infection was much lower in MyD88^−/−^ and TLR7^−/−^ mice (**Figure 5B**, *P*<0.05 or *P*<0.01). Among splenic γδ T cells, Vγ1^+^ subsets in wild-type mice increased significantly at days 3 and 5 post-infection. The magnitude of Vγ1^+^ T cell expansion in MyD88^−/−^ or TLR7^−/−^ mice was reduced at day 3 and remained lower in MyD88^−/−^ mice at day 5 post-infection (**Figure 5C**, *P*<0.05 or *P*<0.01). No changes were observed on Vγ4^+^ T cells in these mice following infection (**Figure 5D**, *P*>0.05). Furthermore, treatment with R848–a TLR7 agonist reduced viral load and increased host survival following WNV infection (Xie G. and Wang T. et al. manuscript in preparation). Here, we infected mice with the WNV NS4B-P38G mutant following i.p. injection with R848. As shown in **Figure 5E**, R848-treated mice had more γδ T cell expansion than did the control group, as analyzed by both percentage among splenic T cells (**Figure 5F**, *P*<0.05) and the total cell number (*P*<0.01). In further phenotypic analysis of γδ T cells in these mice, we found that R848 treatment increased the expansion of Vγ1^+^ T cells, but not Vγ4^+^ T cells (**Figure 5G**, P<0.01). In summary, these results suggest to us that TLR7-MyD88 signaling is involved in the expansion of γδ T cells in particular, Vγ1^+^ T cells during WNV infection.

## Discussion

Besides TCR, γδ T cells express different types of TLRs [19]–[22]. It is known that the TLR-mediated signaling pathways are indirectly involved in γδ T cell activation in mice and humans, mainly via cross- talk with DCs [24]–[27]. Others also reported that TLR ligands co-stimulated IFN-γ and chemokine secretion in TCR-activated human γδ T cells [28], [29]. In this study, we have identified TLR2 and TLR7 as co-stimulating factors during *in vitro* TCR-activation of murine γδ T cells in inducing CD69 expression and Th-1-type cytokine production. Increasing evidence suggests that TLR-mediated signaling pathways alter with aging [40], [48]. One early study by Colonna-Romano et al. showed that γδ T cells from old people and centenarians with enhanced levels of CD69 both after culture in medium alone and in TLR ligand-stimulated cells [49]. Here, we found that TLR2 and TLR7 agonists failed to induce higher CD69 expression and produced less Th-1 type cytokines upon anti-CD3 stimulation of aged γδ T cells compared to young γδ T cells. In addition, increased levels of CD69 expression were noted on γδ T cells of aged mice after culture in medium alone. One possibility is that aging is often associated with increasing levels of both proinflammatory cytokine and regulatory cytokines, like IL-10 and TGF-β [50], [51]. Nevertheless, we found TGF-β levels unchanged after treatment. The magnitude of induction of IL-10 by PAM and CL097 was also reduced on aged γδ T cells after anti-CD3 treatment. The dysregulation of TLR signaling has been associated with impaired functions of monocytes, DCs, and macrophages with aging [52]. Here, our results indicate an impaired TLR signaling contributes to the dysfunction of γδ T cells in aged mice.

The Vγ4^+^ subset is a subpopulation of splenic γδ T cells. CL097 induced more IFN-γ production from non-Vγ4^+^ γδ T cells (most Vγ1^+^ T cells) compared to total splenic γδ T cells. One possibility is that Vγ4^+^ γδ T cells may exert a regulatory role on Vγ1^+^ T cells. Murine Vγ1^+^ and Vγ4^+^ T cells were reported to regulate each other's activity via secreting Th2 and regulatory cytokines [36], [53]. Nevertheless, we noted that there were more IL-4 and IL-10 induced by CL097 upon anti-CD3 treatment on non-Vγ4^+^ T cells. Furthermore, while we noted PAM had the same effect in activating Vγ4^+^ T cells and non- Vγ4^+^ γδ T cells, there was less IL-4 induction by PAM on anti-CD3 treated non-Vγ4^+^ T cells than control group. It seems to be unlikely that these cytokines contribute to a reduced costimulatory effect of CL097. Moreover, we found that TLR7 expression was higher on non-Vγ4^+^ T cells than on Vγ4^+^ T cells. Thus, the differences in TLR7 expression among splenic γδ T cell subsets lead to a differential co-stimulatory effect of TLR7 ligand upon TCR activation. γδ T cells are also the major producer of IL-17 during the early stage of some microbial infection [54], [55]. It is known that distinct γδ T cell subpopulations are committed to produce IFN-γ and IL-17 [56]. In particular, Vγ4 T cell-producing IL-17 contributes to the exacerbation of many diseases, such as collagen-induced arthritis [57], autoimmune encephalomyelitis [55] or psoriasis [58]. Interestingly, the MyD88-dependent TLRs, including TLR2 or 4, are required for the IL-17A response of Vγ4 T cells [59], [60]. Therefore, we conclude that the co-stimulatory effects of TLR ligands on γδ subsets may vary depending on induced cytokine profile and/or TLR expression levels.

The underlying mechanisms of γδ T cell activation during microbial infection are not clearly understood. Previous studies showed that γδ T cell activation in response to *Borrelia burgdorferi* infection in a TLR2-dependent manner, suggesting an involvement of MyD88 signaling during *in vivo* activation [25], [61]. The TLR7-mediated signaling pathway is known to protect the host from lethal WNV infection mainly by promoting IL- 23-dependent immune cell infiltration and homing to the central nervous system [32]. In this study, our results suggest TLR7/MyD88 signaling pathways are involved in γδ T cell activation during WNV infection. Among splenic γδ T cells, Vγ1^+^ but not Vγ4^+^ population, expanded and were activated quickly in response to WNV infection [37]. We have previously shown that Vγ4^+^ T cell- depleted mice had a higher expansion of Vγ1^+^ T cells and were more resistant to WNV infection [36]. Here, we reported that TLR7 signaling preferentially mediated Vγ1^+^ T cell expansion during WNV infection and had a stronger co-stimulatory effect on IFN-γ- induction from Vγ1^+^ T cells upon TCR activation. This is mainly due to a higher expression of TLR7 on this subset. Moreover, Vγ1^+^ T cells of aged mice exhibited a slower and reduced response to WNV infection, which partially contributes to the higher susceptibility to WNV encephalitis [37]. Results from this study now suggest that the reduced effector functions of γδ T cells could be due to the dysregulation of TLR signaling pathways in aged mice. γδ T cell expansion and activation is an important event in host immunity during WNV infection, which is involved in many protective activities, including controlling WNV dissemination and facilitating memory T cell development [46], [62]. Here, we have mainly focused on studying the IFN-γ-producing activity of γδ T cells as it was known to play a predominant role in host protection against lethal WNV infection. For example, adoptive transfer of the splenocytes from TCRβ^−/−^IFNγ^−/−^ mice, which have a defect in the IFN-γ-producing capacity of γδ T cells, did not affect host susceptibility in TCRδ^−/−^ mice [46]. Further, irradiated mice reconstituted with IFN-γ-deficient γδ T cells had enhanced levels of viral loads in blood and brain during WNV infection compared to mice reconstituted with IFN-γ sufficient γδ T cells [63]. The cytolytic function is another important mechanism of viral control attributed to γδ T cells. Future studies will also be focused on investigation of innate immune factors regulating CTL activity during WNV infection. Overall, our data suggests that the MyD88-dependent TLRs are required for the activation and expansion of γδ T cells during microbial infection. γδ T cells are known to form a unique link between innate and adaptive immunity. Due to their unique role in host immunity, understanding of the underlying mechanisms of γδ T cell activation in response to pathogen infection may provide important insights into immunotherapy and vaccine development.

## Supporting Information

Figure S1 **The effects of LPS on anti-CD3- activated γδ T cells of TLR4^−/−^ mice.** *S*plenic γδ T cells were cultured with anti-CD3 with or without LPS. Cells were harvested at 48 h post-stimulation and analyzed for CD69 expression (*A*) and the production of IFN-γ (*B*), IL-2 (*C*) and TNF-α (*D*) in culture supernatant. ** *P*<0.01 or * *P*<0.05 compared to anti-CD3- treated alone. Results presented are one representative of two similar experiments.
(TIF)
