# Supplementary figures and images for: The Co-Stimulatory Effects of MyD88-Dependent Toll-Like Receptor Signaling on Activation of Murine γδ T Cells
Source: PLoS One. 2014 Sep 18;9(9):e108156. doi: 10.1371/journal.pone.0108156 (PMC4169491; doi:10.1371/journal.pone.0108156)

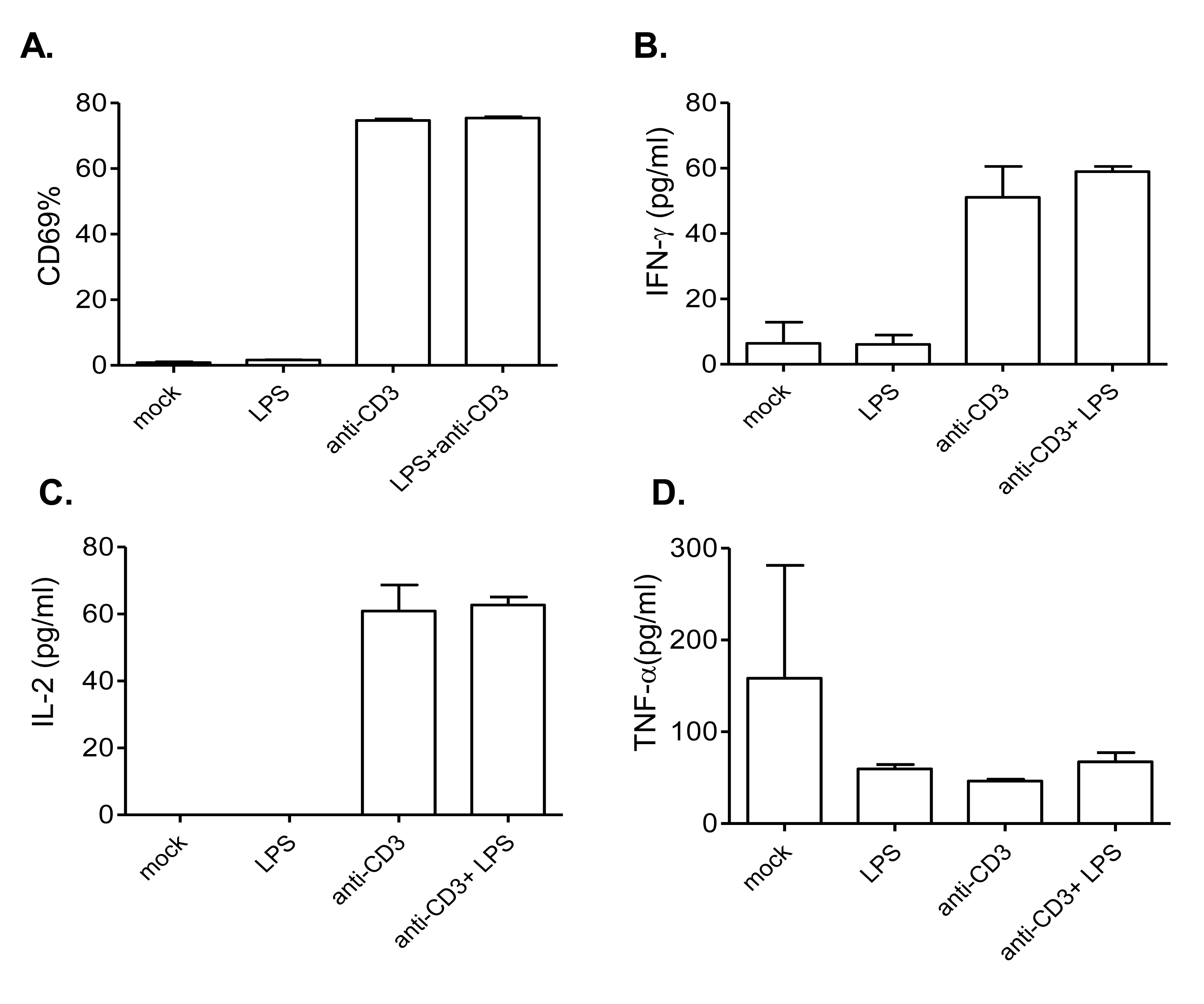

Supplement: Figure S1 — The effects of LPS on anti-CD3- activated γδ T cells of TLR4−/− mice. Splenic γδ T cells were cultured with anti-CD3 with or without LPS. Cells were harvested at 48 h post-stimulation and analyzed for CD69 expression (A) and the production of IFN-γ (B), IL-2 (C) and TNF-α (D) in culture supernatant. ** P<0.01 or * P<0.05 compared to anti-CD3- treated alone. Results presented are one representative of two similar experiments. (TIF) [file pone.0108156.s001.tif]
